# Labor Unions and Staff Turnover in US Nursing Homes

**DOI:** 10.1001/jamanetworkopen.2023.37898

**Published:** 2023-10-13

**Authors:** Adam Dean, Jamie McCallum, Atheendar Venkataramani, David Michaels

**Affiliations:** 1 Deparment of Political Science, George Washington University, Washington, DC; 2 Department of Sociology, Middlebury College, Middlebury, Vermont; 3 Department of Medical Ethics and Health Policy, University of Pennsylvania, Philadelphia; 4 Deparment of Environmental and Occupational Health, Milken Institute School of Public Health, George Washington University, Washington, DC

## Abstract

This cross-sectional study examines the association between labor unions and health care staff turnover in the US using data from 2021.

## Introduction

In an effort to improve nursing home care quality, the federal government recently proposed a new staffing minimum of 0.55 hours per resident day for registered nurses and 2.45 hours per resident day for nurse aides.^1,2,3^ Nursing homes represented by the American Health Care Association argue that high rates of staff turnover would make it difficult for many employers to comply with this potential requirement.^4^ Labor unions representing nursing home workers, such as the Service Employees International Union (SEIU), argue that unions can decrease turnover by improving job quality, thus helping to maintain a stable workforce and improving resident care.^5^ Unionized health care workers earn higher incomes than their nonunionized peers; however, we know little about the association of unions and health care staff turnover.^6^

## Methods

This cross-sectional study followed the Strengthening the Reporting of Observational Studies in Epidemiology (STROBE) reporting guideline. Per the Common Rule, this study was exempt from institutional review board approval and the requirement for informed consent.

We used cross-sectional regression analysis to estimate the association between the presence of a health care workers union and total nursing staff turnover rates in US nursing homes. We focused on 2021, the only year for which data on staff turnover are available from the US Centers for Medicare & Medicaid Services (CMS). We obtained proprietary data from the SEIU on whether a union represented any workers in each nursing home. We adjusted for state fixed effects, county-level population and COVID-19 infection rates, facility-level profit and chain status, star rating, percentage of residents supported by Medicaid, occupancy, staff size, and staffing ratios for registered nurses, certified nursing assistants, and licensed practical nurses. These covariates are publicly available from CMS and LTCFocus. We adjusted for the percentage of nursing home staff who are Black individuals because workplace discrimination against these workers may increase staff turnover. We computed 95% CIs derived from standard errors clustered at the county level.

Unions may be more likely to decrease staff turnover when most nursing homes in the surrounding area are unionized. Such industry-wide unionization enables management to meet union demands without worrying about competition from nonunion facilities with lower labor costs. Therefore, we examined whether the association between facility-level union status and staff turnover varied nonlinearly with the proportion of nursing homes in a county that have a union. For sensitivity tests, we also estimated an unadjusted model and models with alternative covariates, as described in the eMethods in Supplement 1.

## Results

Among 12 633 nursing homes with total nursing staff turnover data available, the mean (SD) staff turnover rate was 52.6% (15.5%). After excluding 1216 facilities with missing data on covariates, 11 417 nursing homes (1971 [17.3%] with unions) were included in our analyses. We found that the presence of a union was associated with a 1.7 percentage point decrease in staff turnover (95% CI, −2.72 to −0.63 percentage points; *P* = .002) (Table). We also found that this association was significantly larger when the county-level proportion of nursing homes that were unionized was high (Figure). When more than 75% of nursing homes in a county were unionized, the facility-level presence of a union was associated with a 9.0 percentage point decrease in staff turnover (95% CI, −15.50 to −2.56 percentage points; *P* = .006) (Figure). When 75% or fewer nursing homes in a county were unionized, a union was associated with a 1.2 percentage point decrease in staff turnover (95% CI, −2.32 to −0.16 percentage points; *P* = .02) (Figure). Our results were robust to the sensitivity tests described in the eMethods in Supplement 1.

**Table.** Association Between the Presence of Health Care Worker Unions and Total Nursing Staff Turnover in Nursing Homes in the United States, 2021

Characteristic	Difference in total nursing staff turnover (N = 11 417)^a^	
	Coefficient	SE (95% CI)
Union^b^	−1.68	0.53 (−2.72 to −0.63)
Number of staff^c^	−0.05	0 (−0.06 to −0.04)
For-profit^d^	2.79	0.20 (2.05 to 3.54)
Chain^d^	2.54	0.31 (1.92 to 3.15)
2-star^e^	−3.27	0.53 (−4.31 to −2.23)
3-star^e^	−5.55	0.51 (−6.54 to −4.55)
4-star^e^	−7.96	0.50 (−8.94 to −6.98)
5-star^e^	−10.70	0.53 (−11.74 to −9.67)
Medicaid, %^f^	−0.02	0.01 (−0.03 to −0.00)
Black staff, %^g^	−0.06	0.02 (−0.11 to −0.01)
Occupancy rate^h^	0	0.01 (−0.02 to 0.02)
Registered nurse staff ratio^i^	−1.16	0.48 (−2.09 to −0.23)
Certified nursing assistant staff ratio^i^	−0.49	0.24 (−0.96 to −0.02)
Licensed practical nurse staff ratio^i^	1.37	0.53 (0.33 to 2.41)
COVID-19 infection rate^j^	−0.25	0.12 (−0.49 to −0.01)
Population^k^	0.30	0.20 (−0.09 to 0.68)

^a^ Coefficient estimates based on ordinary least squares regression adjusted for state fixed effects. The 95% CI were calculated with standard errors clustered at the county level.

^b^ Union representation from Service Employees International Union; binary measure for 2021. Labor unions represent different types of workers throughout different nursing homes, including certified nursing assistants, registered nurses, dietitians, maintenance workers, and other staff.

^c^ Number of nursing home staff from the US Centers for Medicare & Medicaid Services; continuous measure for 2021.

^d^ Ownership characteristics from Brown University’s LTCfocus; binary measures for 2017.

^e^ Star quality rating from the US Centers for Medicare & Medicaid Services; binary measures for 2021.

^f^ Percentage of residents whose primary support comes from Medicaid, from Brown University’s LTCfocus; continuous measure for 2017.

^g^ Percentage of nursing home staff who are Black individuals; continuous measure for 2017 (eReference in Supplement 1). These are results from regression analysis, which adjusted for the percentage of staff who are Black individuals; no other races or ethnicities were adjusted for in this analysis.

^h^ Mean number of residents per 100 certified beds using data from US Centers for Medicare & Medicaid Services; continuous measure 2017.

^i^ Staff hours per resident day from Brown University’s LTCfocus; continuous measure for 2017.

^j^ COVID-19 infections per capita from June 8, 2020, through March 21, 2021.

^k^ County population (log transformed) from USAFacts; continuous measure for 2021.

**Figure.**
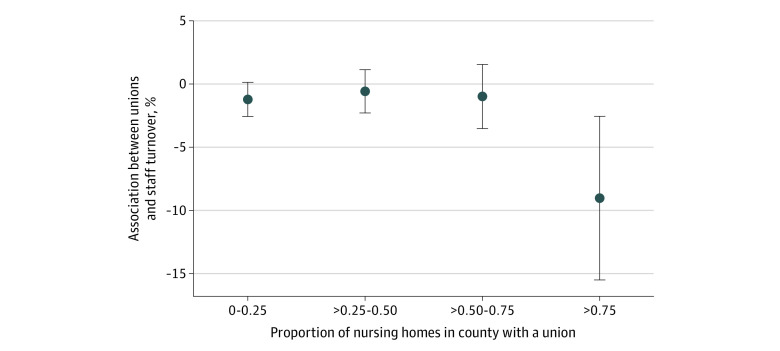
Association Between the Presence of Health Care Worker Unions and Total Nursing Staff Turnover in Nursing Homes at Different Levels of County-Wide Nursing Home Unionization Results based on ordinary least squares regression and a model that includes all covariates from Table 1, as well as interactions between each covariate and 3 different binary variables that take the value of 1 when the county-level proportion of nursing homes with a union is more than 0.25 to 0.50, more than 0.50 to 0.75, and more than 0.75, respectively. The 3 coefficients corresponding to county-level unionization rates of 0 to 0.25, more than 0.25 to 0.50, and more than 0.50 to 0.75 are jointly statistically significant. An additional model that includes all covariates from Table 1, as well as interactions between each covariate and a binary variable that takes the value of 1 when the county-level proportion of nursing homes with a union is more than 0.75 was estimated. The 95% CIs for these models were calculated with standard errors clustered at the county level.

## Discussion

These findings suggest that unions may decrease nursing staff turnover in nursing homes, thus helping employers meet new federal staffing minimums, especially in counties where most nursing homes are unionized. Our study had limitations. First, staff turnover data were only available for 2021. Cross-sectional analysis of these data prevented causal interpretations, and our findings may not generalize outside of the COVID-19 pandemic. Second, union status was only available as a binary measure, precluding analysis of how turnover varied with the percentage or type of workers who are unionized. Third, more research is needed on the mechanisms through which labor unions may reduce turnover, such as increasing wages and improving working conditions.

## Notes

Supplement 1.
eMethods.

eReferences.

Supplement 2.
Data Sharing Statement
