# Supplementary material for: Labor Unions and Staff Turnover in US Nursing Homes
Source: JAMA Netw Open. 2023 Oct 13;6(10):e2337898. doi: 10.1001/jamanetworkopen.2023.37898 (PMC10576215; doi:10.1001/jamanetworkopen.2023.37898)
Supplement: Supplement 1. — eMethods. eReferences. [file jamanetwopen-e2337898-s001.pdf]

Supplemental Online Content

Dean A, McCallum J, Venkataramani A, Michaels D. Labor unions and staff turnover in US nursing homes. *JAMA Netw Open*. 2023;6(10):e2337898.
doi:10.1001/jamanetworkopen.2023.37898

eMethods.
eReferences.

This supplemental material has been provided by the authors to give readers additional information about their work.

© 2023 Dean A et al. *JAMA Network Open.*

eMethods

We assessed the robustness of our findings regarding total nursing staff turnover to two sensitivity analyses. First, we estimated an unadjusted regression model using all 12,633 nursing homes for which data are available on total nursing staff turnover in 2021. Second, we estimated our main model using reduced study cohorts for which more recent measures of our nursing home-level covariates are available.

Sensitivity Test 1

We estimated an unadjusted regression model using all 12,633 nursing homes for which staff turnover data are available.

Sensitivity Test 2

The LTCFocus data used in our main model for nursing home-level profit and chain status, occupancy rate, percentage of residents supported by Medicaid, and staffing ratios (RN, LPN, CNA) were all measured in 2017. We used 2017 data from LTCFocus because of high levels of missing data in more recent releases of this data set. For example, the LTCFocus data for 2020 (the most recent release) only has profit and chain status for 9,712 nursing homes and occupancy rates for 5,668 nursing homes. The 2020 LTCFocus data contains staffing ratios for zero nursing homes. There are similar problems with missing data for our covariates in the LTCFocus data for 2018 and 2019.

© 2023 Dean A et al. *JAMA Network Open.*

As sensitivity tests, we re-estimated our main model using smaller study cohorts and data for different nursing home covariates measured in 2020. First, we revised our main model to include the 2020 LTCFocus data on nursing home profit and chain status. Second, we revised our main model to include the 2020 LTCFocus data on nursing home profit and chain status, as well as occupancy rate and the percentage of residents supported by Medicaid.

eReferences.

1. Shen K. Relationship between nursing home COVID-19 outbreaks and staff neighborhood characteristics. *PLoS One*. 2022;17(4):e0267377. Published 2022 Apr 19. doi:10.1371/journal.pone.0267377

© 2023 Dean A et al. *JAMA Network Open.*
