# Supplementary material for: Labor Unions and Staff Turnover in US Nursing Homes
Source: JAMA Netw Open. 2023 Oct 13;6(10):e2337898. doi: 10.1001/jamanetworkopen.2023.37898 (PMC10576215; doi:10.1001/jamanetworkopen.2023.37898)
Supplement: Supplement 2. — Data Sharing Statement [file jamanetwopen-e2337898-s002.pdf]

Data Sharing Statement

Data
Data available: No

Additional Information
Explanation for why data not available: We obtained proprietary data on nursing home union status from the SEIU and we are not able to publicly share these data.
